# The Role of Bacterial-Derived Aromatic Amino Acids Metabolites Relevant in Autism Spectrum Disorders: A Comprehensive Review

**DOI:** 10.3389/fnins.2021.738220

**Published:** 2021-10-21

**Authors:** Yuanpeng Zheng, Marie K. Bek, Naika Z. Prince, Lucia N. Peralta Marzal, Johan Garssen, Paula Perez Pardo, Aletta D. Kraneveld

**Affiliations:** ^1^Division of Pharmacology, Utrecht Institute for Pharmaceutical Sciences, Faculty of Science, Utrecht University, Utrecht, Netherlands; ^2^Global Centre of Excellence Immunology, Danone Nutricia Research, Utrecht, Netherlands

**Keywords:** autism, gut–brain axis, bacterial metabolites, *p*-cresol sulfate, 4-ethylphenyl sulfate

## Abstract

In recent years, the idea of the gut microbiota being involved in the pathogenesis of autism spectrum disorders (ASD) has attracted attention through numerous studies. Many of these studies report microbial dysregulation in the gut and feces of autistic patients and in ASD animal models. The host microbiota plays a large role in metabolism of ingested foods, and through the production of a range of metabolites it may be involved in neurodevelopmental disorders such as ASD. Two specific microbiota-derived host metabolites, *p*-cresol sulfate and 4-ethylphenyl sulfate, have been associated with ASD in both patients and animal models. These metabolites originate from bacterially produced *p*-cresol and 4-ethylphenol, respectively. *p*-Cresol and 4-ethylphenol are produced through aromatic amino acid fermentation by a range of commensal bacteria, most notably bacteria from the *Clostridioides* genus, which are among the dysregulated bacteria frequently detected in ASD patients. Once produced, these metabolites are suggested to enter the bloodstream, pass the blood–brain-barrier and affect microglial cells in the central nervous system, possibly affecting processes like neuroinflammation and microglial phagocytosis. This review describes the current knowledge of microbial dysbiosis in ASD and elaborates on the relevance and synthesis pathways of two specific ASD-associated metabolites that may form a link between the microbiota and the brain in autism. While the two discussed metabolites are promising candidates for biomarkers and (nutritional) intervention targets, more research into the role of these metabolites in ASD is required to causally connect these metabolites to ASD pathophysiology.

## Introduction

Autism spectrum disorders (ASD) consist of a set of heterogeneous neurodevelopmental conditions, characterized by early-onset deficits in social communication and interaction, and unusually restrictive or repetitive behavior and interests (Lai et al., 2014). According to data from the Autism and Developmental Disabilities Monitoring network from the United States Centers for Disease Control and Prevention (CDC), approximately 1 in 54 children has been identified with ASD (Baio et al., 2018). A recent, large-scale European study estimated an average prevalence of 1 in 89 children in Europe having ASD (Posada de la Paz, 2018). Fombonne et al. (2021) have recently estimated the current worldwide ASD prevalence is 1% based on a thorough epidemiological review, this makes ASD one of the most frequently occurring neurodevelopmental disorders in childhood. The past decades have seen a vast increase in ASD cases, most likely due to changes in the definitions, diagnostic criteria, and increased awareness of ASD, however, a true increase in ASD incidence currently cannot be ruled out (Fombonne, 2009).

Autism diagnosis currently relies on behavioral evaluations, therefore there is a need for valid and clinically useful biomarkers (Lai et al., 2014). Biomarkers can aid diagnosis and can be used to validate effectiveness of interventions (Walsh et al., 2011). Currently, there are no pharmacological treatments targeting the core symptoms of ASD, but behavioral therapies are common practice (Reichow et al., 2012). Various psychological and educational interventions are used to address the behavioral and functional deficits that are associated with ASD (Lai et al., 2014). While no pharmacotherapies addressing the disorder itself exist, two antipsychotic drugs (risperidone and aripiprazole) are approved for the treatment of ASD-associated irritability and aggression (Leskovec et al., 2008).

The pathophysiology of ASD remains elusive, but it is thought to be caused by an interplay between genetic and environmental factors (Chaste and Leboyer, 2012). The disorder is highly heritable and a wide range of susceptibility genes have been identified, accounting for 10–20% of ASD cases (Geschwind, 2011). Possible environmental factors include both pre- and postnatal factors such as prenatal maternal exposure to certain medications, toxins or infections, epigenetic influences, and immune abnormalities (Chaste and Leboyer, 2012). In addition to systemic immune dysregulation, abnormalities in neuroimmune function have frequently been associated with ASD (Onore et al., 2012). The state of chronic neuroinflammation often observed in ASD, is characterized by increased levels of pro-inflammatory cytokines and chemokines in the cerebrospinal fluid (CSF) and activation of microglial cells in the brain tissues of autistic patients (Vargas et al., 2005; Onore et al., 2012).

Gastrointestinal problems, including abdominal pain, diarrhea, chronic constipation, and gastro-esophageal reflux occur frequently in ASD patients (Buie et al., 2010; McElhanon et al., 2014). Possibly related to such intestinal problems, are the changes in composition and activity of intestinal bacteria in ASD, which has now been reported by multiple studies (Finegold et al., 2002; Adams et al., 2011; Iglesias-Vázquez et al., 2020; Roussin et al., 2020). This is in line with the recognition of the intestinal microbiota and its metabolites as a player in neurodevelopmental disorders (Cryan and Dinan, 2012). Moreover, increased intestinal permeability is linked to intestinal dysbiosis in ASD, and may present a potential route for intestinal metabolites into systemic circulation (Hsiao et al., 2013). In addition, several studies have suggested that the severity of GI symptoms is associated with ASD symptoms severity in ASD children (Nikolov et al., 2009; Gorrindo et al., 2012; Mazurek et al., 2013). These findings link the gut microbiota, brain, and behavior together in the form of a microbiota–gut–brain axis in ASD.

As the intestinal microbiota and its metabolites are emerging as important environmental factors in ASD, this report is intended to provide an overview of the current understanding of ASD gut microbial composition and its contribution to the production of two specific ASD-associated bacterial metabolites: 4-methylphenyl sulfate (*p*-cresyl sulfate or *p*CS) and 4-ethylphenyl sulfate (4EPS). Furthermore, the aim of this review is to answer the question of how these ASD-associated metabolites are produced and how they may be involved in ASD pathophysiology.

## Composition of the Gut Microbiota in Autism Spectrum Disorder

### Human Autism Spectrum Disorder Studies

As with the disorder itself, findings with regards to microbial dysregulation in ASD patients are highly heterogeneous. There is currently no consensus on the composition of an ASD-specific microbiota, but some bacterial taxa are frequently reported to have either increased or decreased abundances in ASD patients compared to neurotypical controls.

### *Clostridioides* Bacteria

One of the most frequent and interesting findings is the significantly elevated levels of *Clostridioides* species in fecal samples from ASD patients (Finegold et al., 2002; De Angelis et al., 2013; Iglesias-Vázquez et al., 2020; Kandeel et al., 2020). Bacteria from this genus are suggested to be associated with autism in various ways. Already Bolte (1998) suggested a possible role for *Clostridia* in ASD through tetanus neurotoxin (TeNT) release by *C. tetani*, and subsequent transport of TeNT to the central nervous system (CNS). In addition to TeNT, *Clostridia* are known to produce a range of toxins and potentially toxic metabolites, such as phenols, 4-methylphenol (*para*-cresol or *p*-cresol), and indole derivatives (Finegold et al., 2002).

Another noteworthy association with regards to this genus is the one between ASD and a history of extensive antibiotic use during infancy (Niehus and Lord, 2006). Oral antibiotics can disrupt the protective intestinal microflora and thereby create an environment that is favorable for colonization by opportunistic, toxin-producing bacteria, such as *Clostridioides* species (Bolte, 1998; Sandler et al., 2000). Infection with *C. difficile* and subsequent diarrhea and colitis are associated with broad-spectrum antibiotic therapy, as this bacterium is able to proliferate enterically during use of certain antimicrobials (Kelly and LaMont, 1998). Treatment of *C. difficile* infection involves the specific antibiotics metronidazole or vancomycin (Kelly and LaMont, 1998). The effects of the latter have been investigated in a group of patients with regressive ASD, in which prior use of broad spectrum antibiotics was followed by the development of chronic diarrhea and the manifestation of autistic features (Sandler et al., 2000). Treatment with vancomycin improved autistic symptoms in these patients, however, after treatment discontinuation, the benefits and symptom improvements disappeared. A possible explanation for this relapse is the presence of clostridial spores, which are resistant to antimicrobial agents and allow for clostridial recolonization of the gut (Finegold, 2008). While vancomycin is not feasible as a treatment strategy for ASD, this study further underlines the existence of a connection between the gut, intestinal bacteria such as *Clostridia*, and symptoms of ASD.

### Other Abnormalities

In addition to *Clostridia*, a range of other taxa are reported to be increased in the feces of autistic individuals compared to controls. This includes elevated levels of *Lactobacillus* (Adams et al., 2011), *Ruminococcus* (Finegold et al., 2002), and *Bacteroides* (De Angelis et al., 2013). Members of the *Lachnospiraceae* family (e.g., *Roseburia* and *Dorea*) have also been associated with ASD (De Angelis et al., 2013). Finegold et al. (2010) found that *Desulfovibrio* species and *Bacteroides vulgatus* were present in high numbers in autistic patients and suggested that these bacteria could be important contributors to ASD. In line with this, a study in a cohort of autistic children in Slovakia found an association between *Desulfovibrio* species and autism severity (Tomova et al., 2015). Reports of decreased abundances of certain bacteria are also common and include *Prevotella*, *Coprococcus*, and *Veillonellaceae*, all of which are important for carbohydrate fermentation (Kang et al., 2013). Several studies have also found lower relative abundances of *Bifidobacterium* species (Finegold et al., 2010; Adams et al., 2011; Wang et al., 2011; De Angelis et al., 2013).

There are reports of changes in the *Bacteroidetes/Firmicutes* ratio in the stool of autistic children, but these are inconclusive. Finegold et al. (2010) found increased levels of the phylum *Bacteroidetes* in severely autistic patients, while levels of phylum *Firmicutes* were higher for controls. Conversely, another study reported a decrease in the *Bacteroidetes/Firmicutes* ratio in the feces of autistic patients compared to control, which was normalized by probiotic diet supplementation (Tomova et al., 2015). This inconsistency might be related to the severity of ASD and probiotic intervention, the latter never occurred in first study as the ASD subjects have been excluded if they had been on antibiotics or probiotics during the preceding month. Yet another study found an increase in *Firmicutes* and an accompanying decrease in *Bacteroidetes* in autistic patients with gastrointestinal symptoms (Williams et al., 2011). This discrepancy might be attributed to the ASD children with or without gastrointestinal symptoms. Similar discrepancies are found for other bacteria, for example for *Akkermansia* species; one study found a low relative abundance of the mucolytic bacterium *Akkermansia municipalia* in autistic children (Wang et al., 2011), while others reported the genus *Akkermansia* to be present at high levels in autistic subjects (De Angelis et al., 2013; Kang et al., 2013). Obviously, the ratio of female to male in these studies varies very largely. Additionally, one study shows the relative abundance in species level, but the others show the relative abundance of *Akkermansia* in genus level. Such inconsistent findings seem to reflect the current state of this field of research, possibly due to differences among studies or due to the intrinsic heterogeneity of the disorder. More studies with adequate sample sizes and standardized sequencing techniques are required to pinpoint specific bacterial communities or species that are involved in ASD symptoms and possibly pathology.

### Autism Spectrum Disorder Animal Models

Animal models of autism can provide additional insights and information about the intestinal microbiota and its contribution to pathophysiology of ASD. Various animal models exist and show not only behavioral abnormalities, but often also intestinal and microbial changes consistent with ASD in humans (Needham et al., 2018).

### Valproic Acid Model

In mice, *in utero* exposure to the anticonvulsant valproic acid (VPA), leads to developmental and behavioral deficits in offspring that are similar to ASD (Roullet et al., 2010). de Theije et al. (2014) used this model to assess intestinal microbial composition and found altered microbial colonization as well as an intestinal inflammatory phenotype in VPA-exposed mice compared to control. On the phylum level, a decrease in *Bacteroidetes* and an increase in *Firmicutes*, mainly consisting of *Clostridiales*, was found in the VPA-exposed offspring. But Liu et al. (2018) have shown an increase in *Bacteroidetes* in male rats prenatally exposed to VPA compared to control. This difference might be attributed to different host species (Liu et al., 2018). This is interesting in light of the findings with regards to *Clostridioides* species in individuals with autism. Additionally, significant effects were found for *Desulfovibrionales*, which, as stated above, has also been associated with ASD in children (Finegold et al., 2010; de Theije et al., 2014). Furthermore, this study has also shown a significant increase in cecal levels of butyric acid, one of short chain fatty acids, in male offspring with correlation to the affected microbial abundance by prenatally exposure to VPA (de Theije et al., 2014). In addition, in a VPA model in rats it is shown that the changed fecal microbiota and altered metabolic potential is similar to that observed in ASD (Liu et al., 2018). However, more studies are need to measured real changes in microbiota-associated metabolites in these rodent models for ASD.

### BTBR Mice

The intestinal microbiota composition of BTBR mice, an inbred strain with multiple ASD-like behavioral phenotypes, also shows abnormalities compared to control. Golubeva et al. (2017) reported a reduction in the relative abundance of *Bifidobacterium* and *Blautia* species. This was associated with deficient bile acid and tryptophan metabolism in the intestine, gastrointestinal dysfunction, and impaired social interactions in male BTBR mice (Golubeva et al., 2017). *Akkermansia* bacteria were increased, as well as the *Bacteroidetes/Firmicutes* ratio. Both can be matched to findings from autistic children (Finegold et al., 2010; Kang et al., 2013). Furthermore, there was a reduction in *Bifidobacterium* and *Desulfovibrio* in the BTBR mice, with the reduction in *Bifidobacterium* in accordance with the reduction seen in ASD children (Adams et al., 2011; Golubeva et al., 2017). Two other studies with BTBR mice found a decrease in *Bifidobacterium* and an increase in *Akkermansia* as well (Klein et al., 2016; Newell et al., 2016). Exposure of BTBR mice to a high glycemic index diet during pregnancy and after birth induced higher levels both in the brain and blood of phenolsulfate, a tyrosine metabolite of bacterial origin. Other bacterial phenolic amino acid metabolites were also enhanced in high glycemic index diet fed BTBR mice. These metabolic effects were accompanied by reduced social behavior and cognition and enhanced repetitive behavior when compared to low glycemic index diet fed BTBR mice (Currais et al., 2016). Of interest is the recent study demonstrating that i.v. injection of the gut bacteria produced aromatic metabolite *p*-cresol significantly increased ASD-like behavior in BTBR mice (Pascucci et al., 2020). This study demonstrated a possible causal relation between bacteria derived aromatic metabolites and ASD-like behavior. Overall, the gut microbiota composition and activity of these mice are altered compared to control mice, with similar findings among studies. While some differences compared to human microbiota studies exist, BTBR mice appear to present with microbial dysregulation that is fairly representative for observations from human studies so far.

### Maternal Immune Activation

A different approach to modeling autism in mice is through maternal immune activation (MIA). Epidemiological studies have associated maternal infections during late pregnancy with a higher risk of ASD in the child (Zerbo et al., 2015). MIA offspring present with behavioral abnormalities, decreased intestinal barrier integrity, microbiota alterations, and altered serum metabolomic profile, including elevated level of 4EPS, serotonin, indolepyruvate, glycolate, and imidazole propionate in serum (Hsiao et al., 2013). MIA fecal samples differ in bacterial composition from control animals, with the main driver being changes in the diversity, and not overall abundance, of *Clostridia* and *Bacteroidia*. Increased abundance of bacteria from the families *Prevotellaceae*, *Porphyromonadaceae*, and *Lachnospiraceae* were found for MIA offspring, whereas abundances of *Ruminococcaceae*, *Erysipelotrichaceae*, and *Alcaligenaceae* were higher in controls. Hsiao et al. (2013) suggest a pathogenic role for *Lachnospiraceae* and other *Bacteroidal* species in MIA, whereas other taxa may instead provide protective effects (Golubeva et al., 2017). In this study, treatment with *Bacteroides fragilis* improved barrier function and restored levels of *Lachnospiraceae*, *Bacteroidales*, and several metabolites, such as 4EPS, indolepyruvate, glycolate, and imidazole proprionate, as well as ASD-associated behavioral abnormalities in MIA offspring (Hsiao et al., 2013). Pre-conception microbiota transplantation from MIA mice can transfer susceptibility to neurodevelopmental abnormalities to control mice (Lammert et al., 2018). These MIA studies imply that the maternal microbiota may indeed be a risk factor for the development of neurodevelopmental disorders in offspring from mothers subject to immune activation. To what degree these findings translate to humans remains to be studied.

### Fecal Microbiota Transplant

A recent study found that transplanting human gut microbiota from ASD patients to germ free (GF) mice promotes behavioral ASD-like symptoms in these mice (Sharon et al., 2019). Fecal microbiota transplant (FMT) from ASD donors in mice led to impaired social communication and interaction, and increased repetitive behavior in offspring, which was not found for FMT from neurotypical donors. Not only behavior, but also intestinal microbial composition was different for offspring of the mice that had undergone different FMTs. A decrease in *Bacteroidetes*, *Bacteroides*, and *Parabacteroides* was reported for ASD-FMT offspring, with an increase in *Lachnospiraceae*, *Sutterella*, and *Akkermansia* (Sharon et al., 2019). The increase in *Akkermansia* matches with reports from autistic subjects (Kang et al., 2013) and ASD animal models (VPA and BTBR) (de Theije et al., 2014; Golubeva et al., 2017). Interestingly, metabolomic analysis of serum and colon contents of FMT offspring mice indicated different metabolomic profiles for ASD-FMT offspring and control (Sharon et al., 2019). The taurine and 5-aminovaleric acid were found lower in colon contents from ASD-FMT mice. When these two GABA receptor agonists were administered to BTBR mice, ASD-like behaviors improved (Sharon et al., 2019). Recently, Needham et al. (2021) have shown different metabolic profiles in plasma and feces of bacterial phenolic metabolite levels, between ASD children and typical developing controls. Furthermore, FMT from ASD donors with higher 4EPS level in serum and TD donors into male germ-free mice did not result in differences of this specific metabolite in serum (Needham et al., 2021). 4EPS is only produced by bacteria. Hsiao et al. (2013) have previously shown the 4EPS production has a gender difference in both GF mice and SPF mice, it is much higher in female SPF mice than male SPF mice. It cannot be detected in male GF mice while it still presents in female GF mice (Hsiao et al., 2013; Needham et al., 2021). Very recently, Xiao et al. (2021) have shown the fecal microbiota transplantation of ASD children alters microbial community as well as tryptophan and serotonin metabolism in GF mice, but the causality between induced ASD-like behaviors and altered microbial metabolites remains to be further confirmed.

### Perinatal Antibiotic Treatment

Epidemiological studies have revealed that early-life antibiotic exposure can increase the risk of neurodevelopmental later in life (Niehus and Lord, 2006; Atladóttir et al., 2012; Slob et al., 2021). Indeed, perinatal treatment with low dose penicillin induced impaired social behavior in mice, which was associated with changes in gut microbiota composition, increases cytokine expression in frontal cortex, modifies blood–brain barrier (BBB) integrity (Leclercq et al., 2017). More recent studies, revealed that prenatal low dose penicillin exposure led to abnormal social behavior only in the male offspring. The penicillin-induced changes in microbiota composition clustered differently in both sexes (Champagne-Jorgensen et al., 2020). Antibiotic exposed male mice had significant increased bacteria diversity compared to control and female mice, characterized by increases levels of amongst others *Staphylococcus*, *Parabacteroides*, *Enterococcus*, *Streptococcus*, *Dehalobacterium*, and *Blautia*. The control male offspring had significantly more *Prevotella*, *Bacteroides*, and *Mucispirillum*. Low dose penicillin treatment during lactation induced similar social deficits in male mice, which was associated with increased abundance of *Proteobacteria* and *Firmicutes* at the expense of *Bacteroidetes*. Concurrent treatment with the probiotic strain *L. rhamnosus* prevented the behavioral deficits and normalized microbiota composition (Kayyal et al., 2020).

Overall, reports of microbial dysbiosis in ASD, both in human patients and animal models, are abundant and point toward a role for the gut microbiota in the disease. A well-defined ASD-microbiota profile has, however, not been established yet. Bacterial taxa that appear to be of interest in the context of ASD include *Clostridioides*, *Bifidobacterium*, *Bacteroides*, *Lachnospiraceae*, and *Desulfovibrio*. Whatever the exact changes in composition may be, they will inevitably affect gut and serum metabolites with a bacterial origin, which may have further downstream effects involved in ASD pathophysiology.

## Autism Spectrum Disorder-Associated Bacterial Metabolites *p*-Cresyl Sulfate and 4-Ethylphenyl Sulfate

**FIGURE 1:**
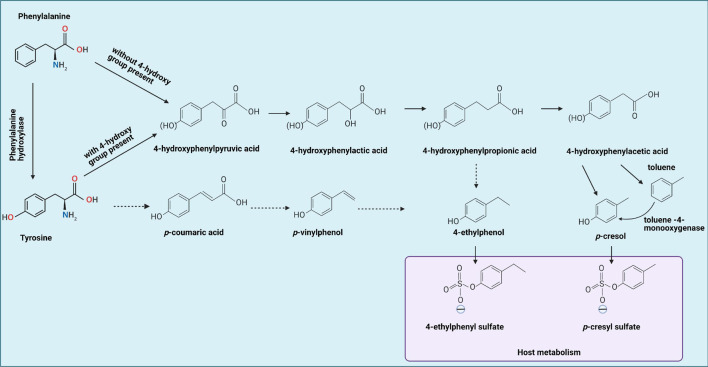
Tyrosine by gut bacteria leading to formation of 4-ethylphenol and *p*-cresol, which can be metabolized by the host into 4-ethylphenyl sulfate and *p*-cresol sulfate, respectively, indicated by the blue box. Solid lines represent established reactions, dotted lines represent presumed reactions taking place. Phenylalanine metabolism occurs similarly, but without the 4-hydroxy group present. In addition, phenylalanine can be converted to tyrosine first, then goes through same procedures with tyrosine metabolism. This figure is adapted from Hsiao et al. (2013), Smith and Macfarlane (1996), and Schuck et al. (2015).

Along with the intestinal composition, the metabolites derived from intestinal bacteria are gaining more attention in ASD research, as various metabolites have been associated with symptoms, severity, or pathophysiology of the disease (Hsiao et al., 2013; Sharon et al., 2019; Xiao et al., 2021). Such microbiota-derived metabolites are *p*-cresol, *p*CS, 4-ethylphenol, and 4EPS. Figure 1 represents an overview of the intestinal microbial and host metabolism involved in the production of *p*-cresol, *p*CS, 4-ethylphenol, and 4EPS (Hsiao et al., 2013; Pascucci et al., 2020). While there are some studies supporting a role for these metabolites in ASD pathophysiology, a clear connection has not been established so far.

### *p*-Cresol and *p*-Cresyl Sulfate

Levels of the bacterial metabolite *p*-cresol and its derivative *p*CS are elevated in urine (Altieri et al., 2011; Gabriele et al., 2014) and feces of autistic children (De Angelis et al., 2013; Kang et al., 2018, 2020). Urinary *p*-cresol has been suggested as a biomarker for autism liability in small children because of its significant elevation (Altieri et al., 2011). Moreover, *p*-cresol has been hypothesized to play a role in ASD pathogenesis (Persico and Napolioni, 2013). The compound is generated by intestinal bacteria, as serum levels of *p*-cresol and its sulfate conjugate *p*CS are significantly decreased in GF mice (Wikoff et al., 2009). Of interest is the recent finding that microbiota transfer therapy lowered enhanced fecal *p*CS levels in ASD similar to those of the typically developing controls (Kang et al., 2020). Furthermore, mice chronically exposed to *p*-cresol in the drinking water demonstrated an ASD-like behavioral phenotype which was shown to be dependent on *p*-cresol-induced changes in the intestinal microbiota composition (Bermudez-Martin et al., 2021). This highlights *p*-cresol and *p*CS as intriguing ASD-associated molecules with possible implications in the microbiota–gut–brain axis in ASD.

Environmental exposure to *p*-cresol through inhalation, skin contact, or food ingestion is also relatively common, with natural sources of exposure including plants, rainwater, petroleum, and tar. Artificial sources of exposure include disinfectants, preservatives, paints, solvents and perfumes, and cosmetics, among others (Persico and Napolioni, 2013). The most important and significant source of *p*-cresol exposure, however, is formed by certain intestinal bacteria that ferment the aromatic amino acids (AAAs) tyrosine and phenylalanine (Persico and Napolioni, 2013).

Once produced by bacteria, 95% of *p*-cresol is metabolized by the host into *p*CS *via* O-sulfonation, a process that occurs primarily in the liver, and to a smaller extent in colonic epithelial cells (Persico and Napolioni, 2013). Approximately 3–4% is metabolized to *p*-cresyl glucuronide and the remaining 1% of *p*-cresol exists in free form in serum and urine (Gabriele et al., 2014), as for the content of *p*-cresol itself in feces or intestine, it remains to be investigated. All three forms are filtered from the blood by glomerular filtration and can be detected at low concentrations in the urine of all individuals (Vanholder et al., 2011). It is not *p*-cresol, but rather its conjugate *p*CS that is more abundant in serum and urine.

Studies reporting on urinary and serum *p*-cresol levels mostly rely on detection methods that first require acidification of the biological sample, which hydrolyzes the conjugates *p*CS and *p*-cresyl glucuronide to *p*-cresol. This means that measured levels of *p*-cresol from biological samples may actually represent levels of *p*CS, and to a lesser extent, *p*-cresyl glucuronide as well as *p*-cresol itself. This indirectly determined *p*-cresol concentration is largely representative of the *p*CS concentration, while it is not responsible for the physiological effects and toxicity that have been attributed to *p*-cresol (Vanholder et al., 2011; Gabriele et al., 2014). In the study that first found elevated levels of *p*-cresol in the urine of autistic children, urine samples were first heated to hydrolyze sulfate and glucuronide conjugates (Altieri et al., 2011), pointing toward an actual increase in *p*CS rather than *p*-cresol. An alternative sample preparation method is methanol deproteinization, which preserves *p*-cresol conjugates, allowing for detection of *p*CS without hydrolysis to *p*-cresol (Cuoghi et al., 2012; Bermudez-Martin et al., 2020). This latter method may present a better method of sample preparation for accurate measurements of *p*CS and *p*-cresol in serum and urine. Currently, there is no standard method of measuring *p*-cresol and *p*CS and therefore concentrations may differ largely among studies.

Urinary *p*-cresol concentration does not correlate with fecal *p*-cresol concentrations, which limits the use of urinary concentrations in making statements about actual intestinal production of *p*-cresol (Birkett et al., 1995). However, urinary concentrations can reflect plasma concentrations of free *p*-cresol and its conjugates (Altieri et al., 2011). Since *p*-cresol is not present at significant concentrations in human serum, physiological effects of *p*-cresol are less relevant than those of *p*CS. However, being produced by bacteria in the colon, the effects of *p*-cresol on intestinal epithelial cells and gut bacteria may still be relevant, as one study has found *p*-cresol to interfere with metabolic activities in colonic epithelial cells, in addition to being genotoxic to these cells (Andriamihaja et al., 2015).

Most of what is known about *p*-cresol and *p*CS originates from their well-established role as toxic retention solutes, as they reach high concentrations in uremic patients where they can exert various toxic effects, for example on the immune system, cardiovascular system, or the brain (Ligabue et al., 2015; Shiba et al., 2018; Lee et al., 2019). In the context of ASD, especially the neurological symptoms occurring in uremic patients are of interest, as uremic toxins such as *p*CS may be involved (Pascucci et al., 2020). Patients with chronic kidney disease are at a larger risk of developing cognitive disorders, dementia, and stroke (Assem et al., 2018). A recent study found high levels of *p*CS and indoxyl sulfate (IS), a related metabolite derived from tryptophan, in the cerebral spinal fluid of patients with Parkinson’s disease and associated these uremic toxins with Parkinson’s pathogenesis and progression (Sankowski et al., 2020). This indicates that uremic toxins may indeed contribute to neurological disorders, but the mechanisms behind a causal connection remain to be established.

Physiological effects of *p*-cresol and *p*CS seem to be almost opposite (Vanholder et al., 2011), so keeping in mind that *p*CS is presumably the largest contributor is important when considering toxicity. Physiological effects of *p*-cresol include decreased endothelial proliferation (Dou et al., 2004), impaired endothelial barrier function (Cerini et al., 2004) and inhibition of leukocyte effector functions *in vitro* through reduced production of reactive oxygen species (Vanholder et al., 1995; De Smet et al., 2003). In contrast, *p*CS can induce free radical producing leukocytes, thereby boosting oxidative stress instead of suppressing it (Schepers et al., 2007). Similar inflammation-inducing effects of *p*CS were found in human monocyte-derived macrophages, in which low concentrations of *p*CS (10, 25 μg/mL; mean uremic concentrations) increased phagocytosis and production of reactive oxygen species (Azevedo et al., 2016). On the other hand, the highest concentration tested in this study (50 μg/mL), corresponding with the maximum uremic concentration, actually decreased the ability to activate the immune cells and initiate a proper immune response to toxins such as lipopolysaccharide (LPS) (Azevedo et al., 2016). Similar suppression of LPS-induced anti-microbial immune responses was observed in *p*CS-exposed murine macrophages (Shiba et al., 2016). Again, especially the highest concentrations of *p*CS (250, 1000 μM) suppressed immune responses by increasing IL-10 and decreasing IL-12 p40. *p*CS can also suppress Th1-type cellular immune responses both *in vitro* and *in vivo*, a mechanism that may be involved in immune dysfunction in patients with chronic kidney disease, as they typically have high concentrations of *p*CS in their blood (Shiba et al., 2014).

It is interesting to expand this hypothesis of microbiota-derived *p*CS being a risk factor for immune dysfunction to include ASD patients, considering the elevated levels of *p*-cresol, and thus *p*CS, that have been found for these patients (Kang et al., 2020). Especially because immune abnormalities are implied in ASD pathophysiology (Onore et al., 2012). The urinary levels of *p*-cresol/*p*CS, found in autistic children are within the range that is found in uremic patients (Gabriele et al., 2014), suggesting that similar effects as those observed in uremic patients could occur in autistic patients as well.

### 4-Ethylphenol and 4-Ethylphenyl Sulfate

Another ASD-associated metabolite is 4EPS, which is structurally related to *p*CS, but is derived from 4-ethylphenol. Like *p*-cresol and *p*CS, 4EPS is derived from, or at least modulated by intestinal bacteria, demonstrated by the finding that germ-free mice have very low serum levels of 4EPS compared to conventionally colonized animals (Hsiao et al., 2013). Elevated concentrations of 4-EPS in serum of children with ASD have been reported (Needham et al., 2021). In a MIA mouse model of autism, serum levels of 4EPS were significantly elevated in autistic-like mice compared to control, and when the animals were treated with the probiotic *B. fragilis*, these elevated levels of 4EPS were fully reduced (Hsiao et al., 2013). This same study showed that systemic 4EPS administration induced anxiety-like behavior in wild-type mice that was comparable to behavior observed in MIA autistic-like mice. While other ASD-characteristic behaviors were not affected, it opens up the possibility of microbiota-derived metabolites playing a role in autism through direct modulation of behavior. Other biological effects of 4EPS have not been studied, and a lot remains unknown about this metabolite. Like *p*CS, it is also a uremic toxin (Kikuchi et al., 2010).

As 4EPS and *p*-cresol have a similar chemical structure, they are thought to have similar effects on the body. However, apart from it being a uremic toxin like *p*CS, little is known about the biological effects of 4EPS. While some studies support the potential relevance of *p*-cresol and *p*CS in ASD, data for 4-ethylphenol and 4EPS is scarce and more studies are required (Needham et al., 2021), for example on toxicity and its effects on the immune system. Further research on these specific ASD-associated bacterial metabolites may elucidate new pathways involved in the disease and possibly present new targets for intervention.

### Aromatic Amino Acid Metabolism

Both *p*CS and 4EPS are products of microbial degradation of AAAs in the gut. With intestinal microbial dysregulation being frequent in ASD patients and models, it is possible that there is a connection between specific intestinal dysregulation and increased levels of metabolites such as *p*CS and 4EPS (Hsiao et al., 2013; Kang et al., 2020). In order to connect these two, it is necessary to first look at which bacterial taxa are involved in the production of *p*-cresol and 4-ethylphenol, the precursors of *p*CS and 4EPS, and then whether these bacteria can be linked to ASD. As *p*CS and 4EPS are sulfonated by the body from *p*-cresol and 4-ethylphenol, respectively, this section will describe the bacterial production of these precursor molecules, both of which originate mainly from bacterial fermentation of the amino acid tyrosine, and to a smaller extent phenylalanine.

Tyrosine and phenylalanine can undergo reductive as well as oxidative metabolism by intestinal bacteria (Dodd et al., 2017). The first step for both processes is an aminotransferase reaction, yielding 4-hydroxyphenylpyruvic acid and phenylpyruvic acid, respectively. Reductive metabolism yields propionic acids: 4-hydroxyphenyl propionic acid (4-HPPA) for tyrosine and phenylpropionic acid for phenylalanine. 4-Ethylphenol can be produced *via* 4-HPPA or *via p*-vinylphenol. Oxidative metabolism yields 4-hydroxyphenylacetic acid (4-HPA) and phenylacetic acid (Dodd et al., 2017), the former being the direct precursor of *p*-cresol. As tyrosine is the main source of *p*-cresol and other hydroxy-phenolic products, the focus will be on tyrosine metabolism (Figure 1).

The production of cresols from tyrosine (and phenylalanine) has been attributed to various intestinal anaerobes, including species of *Clostridioides*, *Bacteroides*, *Bifidobacterium*, and various others (Smith and Macfarlane, 1996; Russell et al., 2013). Through cross-feeding pathways, end products of some species can act as substrates for other species, meaning that even species that do not produce *p*-cresol itself can still contribute to its overall production through synthesis of precursor molecules (Van der Meulen et al., 2008).

### Bacterial *p*-Cresol Production

At least two metabolic pathways for *p*-cresol are known, the first one being oxidation of tyrosine to 4-HPA, which is then decarboxylated to form *p*-cresol (Persico and Napolioni, 2013). Only bacteria that express 4-HPA decarboxylase, such as various species of *Clostridioides* and one genre of *Lactobacillus*, are able to use this pathway (Selmer and Andrei, 2001). The second known synthetic pathway involves oxidation of toluene to *p*-cresol through toluene monooxygenase activity (Whited and Gibson, 1991). Of these two pathways, the one involving tyrosine fermentation by 4-HPA decarboxylase is thought to be more important, as there is much more tyrosine than toluene available as a substrate in the gut lumen and there is a broader distribution of strains that may have the required enzyme for this reaction (Persico and Napolioni, 2013). However, the ability to metabolize tyrosine (and phenylalanine) into *p*-cresol or precursor molecules is not limited to *Clostridioides* and *Lactobacillus* bacteria. This section elaborates on bacteria that may be involved in the production of *p*-cresol from AAAs, and highlights some of them as points of interest for future research into the microbiota–gut–brain axis in ASD. An overview of the bacterial taxa producing *p*-cresol and/or 4-HPA and 4-HPPA is presented in Table 1.

**TABLE 1:** Overview of bacterial taxa producing *p*-cresol and/or 4-hydroxyphenylacetic acid (4-HPA) and 4-hydroxyphenyl propionic acid (4-HPPA).

Family	Genus	Species	*p*-Cresol	4-HPA or 4-HPPA	References
*Clostridiaceae*	*Clostridioides*	*C. bartletti*	X	X	Russell et al., 2013
		*C. bifermentans*		X	Smith and Macfarlane, 1996
		*C. butyricum*	X		Bone et al., 1976
		*C. clostridioforme*	X	X	Van der Meulen et al., 2008
			X		Saito et al., 2018
		*C. difficile*	X	X	Elsden et al., 1976; Dawson et al., 2011; Harrison et al., 2020
			X		Saito et al., 2018
		*C. paraputrificum*	X	X	Bone et al., 1976; Smith and Macfarlane, 1996
		*C. perfringens*	X		Smith and Macfarlane, 1996; Van der Meulen et al., 2008
			X		Saito et al., 2018
		*C. saccharolyticum*	X	X	Russell et al., 2013
			X		Saito et al., 2018
		*C. scatologenes*	X	X	Elsden et al., 1976
		*C. septicum*	X	X	Bone et al., 1976; Elsden et al., 1976
		*C. sporogenes*	X	X	Bone et al., 1976; Elsden et al., 1976
	*Peptostreptococcus*	*P. anaerobius*		X	Lambert and Moss, 1980
*Bacteroidaceae*	*Bacteroides*	*B. eggerthii*	X	X	Russell et al., 2013
		*B. fragilis*	X	X	Bone et al., 1976; Smith and Macfarlane, 1996; Van der Meulen et al., 2008; Russell et al., 2013
		*B. ovatus*		X	Smith and Macfarlane, 1996; Russell et al., 2013
			X		Saito et al., 2018
		*B. thetaiotaomicron*	X	X	Smith and Macfarlane, 1996; Van der Meulen et al., 2008; Russell et al., 2013
		*B. uniformis*	X	X	Russell et al., 2013
			X		Saito et al., 2018
*Bifidobacteriaceae*	*Bifidobacterium*	*B. adolescentis*	X	X	Smith and Macfarlane, 1996; Russell et al., 2013
		*B. animalis*		X	Van der Meulen et al., 2008
		*B. bifidum*	X	X	Smith and Macfarlane, 1996
		*B. infantis*	X	X	Smith and Macfarlane, 1996; Russell et al., 2013
			X		Saito et al., 2018
		*B. pseudolongum*	X	X	Smith and Macfarlane, 1996
*Lachnospiraceae*	*Anaerostipes*	*A. caccae*	X	X	Russell et al., 2013
		*A. hadrus*	X	X	Russell et al., 2013
	*Butyrivibrio*	*B. fibrisolvens*	X	X	Russell et al., 2013
	*Roseburia*	*R. intestinalis*	X	X	Russell et al., 2013
		*R. inulinivorans*	X	X	Russell et al., 2013
*Ruminococcaceae*	*Faecalibacterium*	*F. prausnitzii*	X	X	Russell et al., 2013
	*Ruminococcus*	*R. obeum*	X	X	Russell et al., 2013
		*R. torques*	X	X	Russell et al., 2013
			X		Saito et al., 2018
*Eubacteriaceae*	*Eubacterium*	*E. cylindroides*		X	Russell et al., 2013
		*E. hallii*		X	Russell et al., 2013
		*E. rectale*	X	X	Russell et al., 2013
			X		Saito et al., 2018

### Clostridiaceae

Within the family of *Clostridiaceae*, especially species from the genus *Clostridioides* are involved in the metabolism of AAAs. Various species of this genus are involved in *p*-cresol production, either by performing the final decarboxylation step of 4-HPA or by producing precursor molecules of *p*-cresol that may be further metabolized by cross-feeding bacteria. The species that has attracted most attention with regards to *p*-cresol production is *C. difficile*, which expresses 4-HPA decarboxylase in order to catalyze the formation of *p*-cresol (Elsden et al., 1976; Selmer and Andrei, 2001; Harrison et al., 2020). Interestingly, *C. difficile* has a high tolerance for *p*-cresol, whereas *p*-cresol is toxic to many other microbes *via* its ability to interfere with metabolism and inhibit growth (Hafiz and Oakley, 1976; Dawson et al., 2008). This ability to produce and tolerate *p*-cresol is thought to provide the bacterium with a competitive advantage over other intestinal bacteria (Passmore et al., 2018). High *p*-cresol tolerance has also been reported for two other *Clostridioides* species, *C. perfringens* and *C. sordellii*, even though these species produce little to no *p*-cresol themselves. This indicates overlap in tolerance pathways amongst various species of this genus (Dawson et al., 2008, 2011). As discussed earlier, infant antibiotic use has been associated with ASD, which has led to speculations about antibiotics disrupting the protective commensal microflora, thereby creating an environment that allows for colonization by opportunistic bacteria like *Clostridioides* species (Bolte, 1998). This could be accompanied by increased levels of *Clostridioides*-derived *p*-cresol, which in turn could maintain suppression of (parts of) the intestinal microbiota due to high *p*-cresol concentrations.

Production of *p*-cresol is not unique to *C. difficile* as it has also been demonstrated for *C. scatologenes* (Elsden et al., 1976) and other *Clostridioides* species including *C. paraputrificum*, *C. perfringens*, *C. septicum*, and more (Table 1; Bone et al., 1976; Smith and Macfarlane, 1996). Other *Clostridioides* species can metabolize tyrosine and/or phenylalanine to phenolic compounds that can be used by other species to form *p*-cresol. Various molecules that can act as precursors for *p*-cresol can be produced (Figure 1), but especially the production of 4-HPA and 4-HPPA are likely to be contributors to *p*-cresol production further down the line. Species known to produce 4-HPA include *C. difficile* and *C. scatologenes* among others (Table 1; Elsden et al., 1976; Russell et al., 2013). *C. sporogenes*, *C. paraputrificum*, *C. bifermentans*, and *C. septicum* have been found to produce 4-HPPA, which may act as a cross-feed precursor of both *p*-cresol and 4-ethylphenol, which is the precursor of 4EPS (Elsden et al., 1976; Smith and Macfarlane, 1996).

These multiple *Clostridioides* species involved in *p*-cresol production, as well as the associations between *Clostridioides* abundances and ASD, make this genus and its capacity to produce ASD-associated metabolites interesting for future research in ASD. At least it warrants further research into a possible (direct) link between Clostridia and autism, for example by assessing fecal and urinary *p*-cresol and serum *p*CS levels as well as intestinal microbiota composition of autistic patients. Additional behavioral testing can provide further evidence on such an association.

Another genus of *Clostridiaceae* involved in AAA metabolism is *Peptostreptococcus*. *P. anaerobius* can metabolize tyrosine to 4-HPPA through deamination of tyrosine into *p*-coumaric acid and subsequent reduction (Lambert and Moss, 1980). Both products can be used as precursors for *p*-cresol and 4-ethylphenol production (Figure 1). However, *P. anaerobius* does not produce *p*-cresol itself, and thus is presumably only a cross-feeder (Turgeon et al., 1990).

### Bacteroidaceae

In addition to *Clostridioides*, some species of *Bacteroides* are able to ferment AAAs. *B. fragilis*, *B. thetaiotaomicron*, and *B. uniformis* can produce *p*-cresol from tyrosine (Smith and Macfarlane, 1996; Russell et al., 2013). Other species such as *B. ovatus* may contribute to overall *p*-cresol through the production of 4-HPA or 4-HPPA (Table 1). According to Russell et al. (2013) there is a substantial population of AAA-metabolizing *Bacteroides* in the gut of most individuals and the AAA-metabolizing activity of these bacteria is high. Overall, the major metabolite of *Bacteroides* fermentations is phenylacetic acid, but 4-HPA is also produced in significant amounts (Russell et al., 2013). While direct *p*-cresol synthesis has only been proven for a few species of this genus, and only in low concentrations, its contribution to overall *p*-cresol through synthesis of precursor molecules may still be considerable.

Despite this possible contribution to the ASD-associated metabolite *p*-cresol, there is evidence that probiotic treatment with *B. fragilis* or the related *Bacteroides* species *B. thetaiotaomicron* and *B. vulgatus* has beneficial effects on ASD-like symptoms in MIA animal models of ASD (Hsiao et al., 2013; Mazmanian et al., 2016). Probiotic administration of *B. fragilis* ameliorates ASD-like symptoms and normalizes serum levels of 4EPS and other metabolites characteristic for the ASD-like mice, possibly through improvement of intestinal barrier function (Hsiao et al., 2013). So, while some species of this genus can contribute to the production of ASD-associated metabolites, they may also be protective through reducing serum levels of these metabolites. It may depend on the species involved or possibly a range of additional environmental factors.

### Bifidobacteriaceae

Metabolites of tyrosine and phenylalanine have been detected for *Bifidobacterium* fermentations, with (hydroxy)phenyllactic acid being the major metabolite. *B. animalis* can produce hydroxyphenyllactic acid as well as low levels of 4-HPA (Van der Meulen et al., 2008). While this study did not detect *p*-cresol in the fermentations with *Bifidobacteria*, Smith and Macfarlane (1996) earlier reported *p*-cresol production from tyrosine by various species of this genus, including *B. bifidum*, *B. adolescentis*, *B. infantis*, and *B. pseudolongum*. Of the *Bifidobacterium* species, only *B. longum* fermented phenylalanine, thereby producing phenyllactate, and no *p*-cresol or direct precursors, indicating that *p*-cresol is predominantly formed by tyrosine-fermenting bacteria (Smith and Macfarlane, 1996).

*Bifidobacteria* are considered to be beneficial commensal bacteria and have been reported to be decreased in autistic patients compared to control (Adams et al., 2011; Wang et al., 2011). A few trials have assessed the effects of probiotics including *Bifidobacterium* species on behavior and intestinal symptoms in autistic children. Some studies report amelioration of behavior and/or intestinal symptoms, but due to different probiotic combinations and different designs, studies are too heterogeneous to draw conclusions about the real benefit of these bacteria (Ng et al., 2019).

### Lachnospiraceae

One study found that bacteria within the *Lachnospiraceae* family are also able to metabolize tyrosine to *p*-cresol and 4-HPA although concentrations were low (Russell et al., 2013). Within the genera *Anaerostipes*, *Butyrivibrio*, and *Roseburia* there were five species able to produce both *p*-cresol and 4-HPA (Russell et al., 2013). Increased abundances of *Lachnospiraceae* have been associated with ASD both in humans and an animal model of ASD (De Angelis et al., 2013; Hsiao et al., 2013). An *in vivo* study found that social avoidance behavior after microbiota transplantation was accompanied with elevated *Lachnospiraceae* and high levels of *p*-cresol, but the causalities remain to be further confirmed (Gacias et al., 2016). Apart from these findings, information about these bacteria is scarce, but as they are increased in autistic patients and models, and can contribute to production of ASD-associated metabolites, a role in ASD pathophysiology or symptoms is possible and further research could be valuable.

### Ruminococcaceae

Russell et al. (2013) detected low levels of *p*-cresol and 4-HPA from tyrosine fermentation for *Ruminococcus obeum* and *Ruminococcus torques*, as well as for *Faecalibacterium prausnitzii*. In this study, these bacteria produced high amounts of benzoic acid and 4-hydroxybenzoic acid. Phenylpyruvic acid and phenyllactic acid, metabolites of phenylalanine, were also produced in significant amounts by these bacteria. While these molecules are not directly linked to *p*-cresol, contribution to cross-feeding pathways cannot be excluded. A potential association between increases in *Ruminococcaceae* and elevated *p*-cresol levels might be made with regards to social avoidance behavior in mice (Gacias et al., 2016), if further direct evidences show this family can produce *p*-cresol in the mice gut.

### Eubacteriaceae

In their elaborate study on AAA-metabolizing bacteria, Russell et al. (2013) found three *Eubacterium* species could produce phenylacetic acid and 4-HPA from phenylalanine and tyrosine, respectively (Table 1). Especially *E. hallii* was found to produce significant amounts of phenylacetic acid and 4-HPA. *p*-Cresol was detected only for *E. rectale* fermentations, but concentrations were low (Russell et al., 2013). With only one study assessing this family of bacteria, a firm connection cannot be made, but neither can involvement be excluded.

### Bacterial 4-Ethylphenol Production

While multiple studies have investigated the production of *p*-cresol and 4-HPA as end products of bacterial AAA fermentation, less data on 4-ethylphenol, the presumed precursor of 4EPS, is available. Just as the other simple phenols (phenol and *p*-cresol), 4-ethylphenol is thought to originate from microbial metabolism of tyrosine (Drasar and Hill, 1974; Bone et al., 1976). Tyrosine can be reductively deaminated to 4-HPPA, which in turn can be decarboxylated to form 4-ethylphenol (Figure 1; Drasar and Hill, 1974). Through this synthetic pathway, essentially all bacterial taxa that have been found to produce 4-HPPA could be involved in the production of 4-ethylphenol, possibly through cross-feeding pathways as well. However, experimental data in support of this metabolic pathway is scarce. In fact, it has even been opposed by some studies that found that 4-HPPA could only be dehydroxylated to phenylpropionate and that no further metabolism occurred (Martin, 1982; Smith and Macfarlane, 1997).

An alternative synthetic pathway includes the formation of *p*-coumaric acid from tyrosine, which may be decarboxylated to form *p*-vinylphenol and subsequently reduced to 4-ethylphenol (Figure 1). There is limited literature elaborating on this synthetic pathway and whether commensal bacteria are involved. Instead, research on 4-ethylphenol is mainly centered around its production by yeasts in food fermentations (Suárez et al., 2007).

A recent study reported that a whole range of intestinal bacteria can produce phenolic precursors of 4-ethylphenol, including *Coriobacteriaceae*, *Enterobacteriaceae*, *Fusobacteriaceae*, and *Clostridioides* clusters I and XIVa (Saito et al., 2018). Production of 4-ethylphenol by *Clostridial* species has also been suggested in earlier studies (Bone et al., 1976; Nicholson et al., 2012). Findings from the MIA study from Hsiao et al. (2013) support this idea, as both elevated serum levels of 4EPS and increased levels of the *Lachnospiraceae* family of Clostridia were observed in MIA autistic mice. While this is no direct evidence, it indicates an association between bacteria of the *Lachnospiraceae* family and serum 4EPS levels. In the same study, however, it has been shown that treatment of mice with 4EPS indeed resulted in anxiety-like phenotype demonstrating a causal relationship between exposure to 4-EPS and behavioral changes. Moreover, Hsiao et al. (2013) suggest that the structural similarity of 4EPS to the (mainly) *Clostridioides*-derived *p*-cresol and *p*CS could be an indicator of similar biosynthetic pathways, again pointing toward clostridial involvement in the production of 4-ethylphenol and 4EPS.

Recently, Santamaría and coworkers found that *Lactobacillus plantarum* is able to produce 4-ethylphenols (Santamaría et al., 2018). It is worthwhile mentioning that recently a 4-week randomized double-blind placebo-controlled study demonstrated some beneficial effects in ASD of *L. plantarum* PS128 (Liu et al., 2019). These findings may contrast a pathological role of 4-ethylphenol and the associated host metabolite 4EPS in ASD. This calls for further research into the role of 4-ethylphenol and 4EPS and the bacteria involved in their production in ASD.

To conclude, there are various candidates for bacterial *p*-cresol and 4-ethylphenol production, some of which have previously been associated with ASD in animal models or in humans (Hsiao et al., 2013; Kang et al., 2020). The most promising genus for association with *p*-cresol in ASD is *Clostridioides*, as these bacteria are known for their prominent *p*-cresol production and have continuously been hypothesized to play a role in ASD. For the production of 4-ethylphenol and 4EPS, the specific bacteria involved are less clear, but also here clostridial species are candidate bacteria. Future studies assessing both microbial dysregulation and metabolite levels in ASD patients could provide evidence for the existence of a connection between specific dysregulation and levels of *p*CS and 4EPS.

## The Potential Effect of Bacterial Metabolites on Brain in Autism Spectrum Disorder

After being produced by the intestinal microflora, some bacterial metabolites are thought to be able to exert neurotoxic effects on the brain when they are present at high concentrations. An example is the proposed role for TeNT produced by *Clostridia* species in ASD development (Bolte, 1998). In a similar way, *p*CS and 4EPS may travel to the CNS and possibly affect neuroglial cells and thereby processes like neuroinflammation, phagocytosis, and affect synaptic pruning in the CNS. Neuroinflammation is a key finding in autistic patients and is characterized by marked activation of microglial cells and concomitant increased levels of inflammatory cytokines and chemokines in the CSF (Vargas et al., 2005; Morgan et al., 2010; Onore et al., 2012). Microglial cells are a specialized population of macrophages in the CNS. They play a role in innate immune function through release of inflammatory mediators and recognition and subsequent phagocytosis of microorganisms or damaged or infected cells (Boche et al., 2013). Additionally, microglial cells are crucial for neuronal development as they are involved in synaptogenesis and responsible for the pruning of redundant synaptic connections during childhood, a process that is necessary for the development of functional neural circuits (Paolicelli et al., 2011).

As a result of neuroinflammation, there may be changes in the cytokines and chemokines secreted by microglial cells, which may in turn disrupt synapse maintenance. The resulting changes in dendritic branching and spine density possibly contribute to under- and hyper-connectivity in various brain regions frequently observed in autistic patients (Matta et al., 2019). Dysregulations in the process of synaptic pruning can disrupt the excitatory/inhibitory balance of synapses, which may contribute to neurodevelopmental disorders such as ASD (Koyama and Ikegaya, 2015).

### The Potential Effects on Blood–Brain-Barrier Integrity

The metabolites discussed here are postulated to have neurotoxic effects (Finegold et al., 2002), but before assessing whether the metabolites *p*CS and 4EPS affect processes like neuroinflammation in ASD, their ability to reach the brain needs to be established, as transport to the CNS is required for the exertion of such direct effects. In order to reach the brain, the metabolites have to pass the BBB, the border between systemic circulation and the CNS. Results from one *in vivo* study show that *p*CS can indeed reach the brain, as *p*CS was shown to accumulate in the brain in mice with renal failure (Sato et al., 2017). This indicates that *p*CS can cross the BBB in mice with renal failure, where the microbiota is dysregulated and serum levels of *p*CS and other uremic toxins are high, seemingly similar to in individuals with ASD (Lau et al., 2018). To this point, ability to reach the CNS has not been demonstrated directly for 4EPS. However, Hsiao et al. (2013) found that systemic administration of 4EPS led to changes in behavior in mice (e.g., inducing anxiety-like behavior), an effect that can be hypothesized to be caused by 4EPS directly affecting the brain.

**FIGURE 2:**
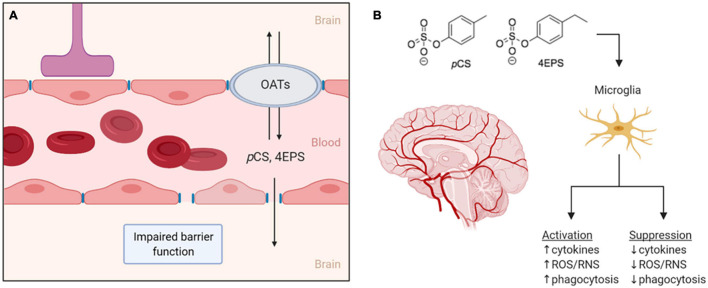
**(A)** Two proposed routes for *p*CS and 4EPS to move from the bloodstream into the brain. The first is through the impaired blood–brain-barrier associated with ASD and the second through organic anion transporters that may be expressed in the blood–brain-barrier. **(B)** If *p*CS and 4EPS pass the blood–brain-barrier and enter the central nervous system, these bacteria-derived metabolites might affect microglial cells, either by activating or suppressing them. OATs, organic anion transporters; *p*CS, *p*-cresyl sulfate; 4EPS, 4-ethylphenyl sulfate; ROS/RNS, reactive oxygen/nitrogen species. Created by BioRender.

There is limited data on *p*CS and 4EPS reaching the brain and the mechanisms facilitating this. Different mechanisms of moving from systemic circulation into the CNS can therefore be proposed. One factor likely to be involved is the impaired integrity and thus increased permeability of the BBB that is associated with ASD (Fiorentino et al., 2016). Abnormalities in endothelial BBB permeability can parallel the ASD-associated abnormalities in the gut microbiota. The microbiota has been identified as a regulator of BBB integrity, as germ-free mice have increased BBB permeability and reduced expression of tight junction proteins that regulate barrier function. These effects are reversed when germ-free animals are colonized with conventional gut microbiota (Braniste et al., 2014). Impaired BBB integrity has also been reported for an animal model of chronic kidney disease (Lau et al., 2020). This may be mediated by urea and other uremic toxins, as brain endothelial barrier function is impaired upon exposure to urea or serum from uremic patients in cell culture studies (Lau et al., 2020). While more data is needed, these findings do suggest a role for (microbiota-derived) uremic toxins in BBB permeability. Due to the similarities in uremic toxins that have been found in uremic patients and individuals with ASD, it could be possible that similar mechanisms are involved in the disrupted BBB integrity in ASD. In turn, this disrupted integrity could facilitate entry of microbiota-derived metabolites into the CNS (Figure 2A).

In general, uptake of uremic toxins into tissues is thought to involve transport though organic anion transporters (OATs) (Sato et al., 2017). OATs are expressed at barrier epithelia in various tissues in both facilitate the transport of a variety of drugs, dietary compounds, and urinary toxins, usually after the products have been modified in some way (e.g., hydroxylated, sulfonated, or glucuronidated) (Nigam, 2018). For transport and clearance of certain microbiota-derived molecules, the expression of OAT1 and OAT3 in the kidneys is especially important, demonstrated by the accumulation of microbiota-derived uremic toxins in OAT1 and OAT3 knockout studies. The accumulating molecules include *p*CS, 4-HPA, and IS, a product from tryptophan metabolism that is similar to *p*CS (Nigam, 2018). OATs, presumably preferentially OAT3, are responsible for the uptake of *p*CS in rat kidneys (Miyamoto et al., 2011). OAT3 is also expressed in the brain of rats, where it is suggested to be involved in molecular transport across the BBB (Kusuhara et al., 1999). IS was found to be transported across the BBB *via* OAT3 in rats (Hosoya and Tachikawa, 2011). This transport by OAT3, however, is effluent, from the brain/CSF to the blood, and thus forms a mechanism of eliminating toxins such as IS from the brain, which is necessary for protection of the brain (Hosoya and Tachikawa, 2011). These findings allow for speculations on whether OATs could provide a way across the BBB for the metabolites, *p*CS and 4EPS, as well, and perhaps facilitate influx and/or efflux of these compounds in the CNS (Figure 2A).

### Effects on Microglial Cells

The importance of the host microbiota in regulating microglial homeostasis is indicated by the finding that germ-free mice have defective microglia and subsequently impaired innate immune responses in the CNS, both of which can be restored by colonization with conventional microbiota (Erny et al., 2015). Microbiota-derived short chain fatty acids seem to be the regulators of microglial homeostasis (Erny et al., 2015), but additional microbiota-derived molecules can be involved too.

Assuming that *p*CS and 4EPS can indeed reach the CNS, it can be hypothesized that both metabolites either increase microglial activation and effector functions or suppress them. Effects of low concentrations of *p*CS on macrophages are pro-inflammatory, shown by increased phagocytosis and reactive oxygen species production, whereas higher concentrations of *p*CS have immunosuppressive effects, resulting in the inability to initiate a proper immune response (Azevedo et al., 2016; Shiba et al., 2016). Thus, based on the limited experimental knowledge available, both options appear to be possible. Increased microglial activation and a subsequent state of neuroinflammation is associated with ASD (Onore et al., 2012), however, the same has been hypothesized for defective microglia with a decrease in microglia-mediated synaptic pruning (Koyama and Ikegaya, 2015). The mechanisms underlying both of these ASD-associated states is not fully understood, and possibly, the ASD-associated metabolites discussed here could play a role.

### Effects on Microglial Phagocytosis

One study investigating microglial density and morphology in postmortem ASD samples, found significantly increased somal volume in microglia from ASD patients compared to control (Morgan et al., 2010). Additional morphological alterations included a reduction in the number of processes extending from the cell bodies as well as a shortening and thickening of these processes. This more amoeboid morphology is characteristic of microglial reactivity and phagocytic activity (Boche et al., 2013). A phagocytic morphology is required in early life for the process of synaptic pruning, however, if such a morphology persists, it can be an indicator of a state of chronic activation. A hypothesis fitting with this scenario is that the bacterial metabolites could play a role in the increased inflammatory activity of microglial cells in which an upregulation of microglial phagocytosis could be expected.

An alternative possibility is that of inducing neuroinflammatory processes, the bacterial metabolites dysregulate or suppress the immune system. Rather than an anti-inflammatory effect, this points toward a dysregulated immune response against toxins such as LPS, which are supposed to initiate a proper immune response in order to protect the brain from damage. An immunosuppressive role is supported by studies finding that *p*CS affected the immune system’s ability to initiate a normal immune response against LPS (Shiba et al., 2014; Azevedo et al., 2016). These immunosuppressive effects of *p*CS and 4EPS may extend to phagocytic capacity of microglia in the CNS. Impaired leukocyte phagocytosis has been observed in uremic patients undergoing dialysis, again strengthening the possibility of a connection between uremic toxins and immune suppression (Alexiewicz et al., 1991). Inhibition of microglial autophagy has been shown to impair degradation of synapses and debris, indicating disturbed synaptic pruning (Kim et al., 2017). Microglial autophagy and phagocytosis are both mechanisms by which the cell can degrade extracellular materials such as pathogens, damaged cells, and surplus synapses. The inhibition of autophagy, and thus synaptic pruning, results in ASD-like social and behavioral defects as well as repetitive behaviors in mice (Kim et al., 2017). Thus, it could also be possible that the bacterial metabolites contribute to ASD pathophysiology through impaired microglial activity and subsequent impaired synaptic pruning. Alternatively, deficient microglia may be damaging to the brain as protection against invading pathogens is no longer guaranteed. This can be especially problematic in combination with impaired BBB integrity, allowing pathogens and toxins to enter the CNS, where a proper immune response cannot be initiated, allowing invading pathogens and toxins to exert their damage on the brain.

Whether and how precisely *p*CS and 4EPS affect microglial phagocytosis and whether this contributes to the pathogenesis of ASD is currently unknown (Figure 2B), and presents an opportunity for future research, as described in the next section.

## Discussion

While a causal role of microbial dysregulation and microbiota-derived metabolites such as *p*CS and 4EPS in ASD has not been confirmed, it is still possible to think about possible therapeutic strategies based upon such a connection between the microbiota, metabolites, and the brain. The idea that probiotics may be beneficial in the context of ASD has been explored by a few studies but study populations are small, heterogeneous and the interventions differ among studies, making it difficult to draw conclusions about the effects of probiotic interventions (Ng et al., 2019). Additionally, studies taking specific metabolites of bacterial AAA fermentation into account are scarce. One preclinical study has shown a beneficial effect of *B. fragilis* treatment in lowering serum levels of 4EPS in a mouse model of autism, but whether this can be translated to humans remains to be studied (Hsiao et al., 2013).

Further research can contribute to the design of a probiotic therapy aimed at normalizing microbial dysregulation associated with altered metabolite levels in ASD patients. For example, if an increase in clostridial species in the gut of autistic patients is found to be directly associated with increased levels of *p*CS and 4EPS, a therapy targeted against clostridial bacteria can be beneficial by lowering metabolite levels and thereby their possibly detrimental effects on the brain and behavior. Possibly, probiotic administration of other species can be used to normalize or prevent dysregulated microbiota. The idea of using probiotic therapies to relieve both intestinal and behavioral abnormalities associated with neurodevelopmental disorders is supported by various studies, for example by the research performed by Buffington et al. (2016). They found a single commensal strain, *Lactobacillus reuteri*, to restore intestinal and social behavior abnormalities associated with Maternal High-Fat Diet offspring (Buffington et al., 2016). Additionally, there is evidence that probiotics are connected to lower risks of *C. difficile* infections (Goldenberg et al., 2018). With clostridial species implied as troublemakers in ASD, such a method to prevent infection and keep specific bacteria under control might also be beneficial in autism. Additionally, probiotic treatment to restore epithelial integrity in the gut may prevent metabolites from entering systemic circulation, which may prevent some complications. There are already indications that probiotics can indeed restore disrupted intestinal barrier integrity (Arseneault-Bréard et al., 2012; Hsiao et al., 2013).

Another interesting approach is targeting the elevated levels of the metabolites directly, instead of the bacteria producing them. An oral sorbent, AST-120, is available for treatment of chronic kidney disease, in which it adsorbs uremic toxins and their precursors within the intestinal tract (Liu and Dreyfuss, 1996). By adsorbing *p*-cresol and 4-ethylphenol in the intestine, the accumulation *p*CS and 4EPS in serum and urine is prevented (Kikuchi et al., 2010). Moreover, one preclinical study showed that *p*CS accumulation in the brain of uremic mice can be reduced to normal by AST-120 (Sato et al., 2017). While this has not been studied in relation to ASD, and it may not be a sustainable method, it does present a way of targeting the metabolites directly. In addition, Pascucci et al. (2020) have shown *p*-cresol administration exacerbates ASD-like behaviors in BTBR mice, which is associated with enhanced dopamine and its metabolites DOPAC and HVA levels in specific brain areas. Previously, it has been shown with *in vitro* studies that *p*-cresol inactivates dopamine β-hydroxylase as an alternative substrate of this enzyme. In this way, *p*-cresol binds to this enzyme competitively and ends up with the inhibition of metabolism dopamine to norepinephrine, resulting in that less dopamine is converted to norepinephrine (Goodhart et al., 1987; DeWolf et al., 1988; Southan et al., 1990).

While the ideas described above are currently merely hypothetical, the idea of such novel targeted therapies is intriguing, especially in light of the current absence of therapies targeting underlying mechanisms of ASD. Even so, in spite of new ideas and promising findings from preclinical studies, the mechanistic and translational knowledge is still lacking. Whether we can connect the different aspects to form a complete story that really contributes to the understanding and possibly treatment of ASD depends on future research.

## Conclusion

The notion that the microbiota is in some way involved in neurodevelopmental disorders such as ASD continues to acquire evidence. As outlined in this review, specific microbiota-derived metabolites may form a link between the microbiota and the brain, and future studies on the effects of such metabolites may provide insights into the pathophysiology and possibly, etiology of ASD. The metabolites highlighted in this report, *p*CS and 4EPS have been associated with ASD in multiple studies, but strong evidence for causal involvement is still missing. These metabolites are produced from fermentation of AAA by a range of intestinal bacteria, most notably bacteria from the *Clostridioides* genus. These bacteria are among the dysregulated bacteria often observed in ASD patients. Future research may elucidate whether these and other metabolite-producing bacteria are indeed involved in ASD. Both *p*CS and 4EPS have been singled out as promising biomarkers and along with the microbiota, as possible targets for novel therapies, either through pharmacological or nutritional interventions targeting the microbiota–gut–brain axis.

## Author Contributions

YZ and MB: literature research and drafting the manuscript. AK and PP: conceptualization and refining the manuscript. NP, LP, and JG: critical reading of the manuscript. All authors have read and approved the manuscript.

## Conflict of Interest

JG is a part time employee at Danone Nutricia Research, Utrecht, Netherlands. The remaining authors declare that the research was conducted in the absence of any commercial or financial relationships that could be construed as a potential conflict of interest.

## Publisher’s Note

All claims expressed in this article are solely those of the authors and do not necessarily represent those of their affiliated organizations, or those of the publisher, the editors and the reviewers. Any product that may be evaluated in this article, or claim that may be made by its manufacturer, is not guaranteed or endorsed by the publisher.
